# Galectin-3 and Blood Group: Binding Properties, Effects on Plasma Levels, and Consequences for Prognostic Performance

**DOI:** 10.3390/ijms24054415

**Published:** 2023-02-23

**Authors:** Carolin Pozder, Elles M. Screever, A. Rogier van der Velde, Herman H. Silljé, Janne Suwijn, Saskia de Rond, Marcus E. Kleber, Graciela Delgado, Jan Jacob Schuringa, Wiek H. van Gilst, Wouter C. Meijers, Winfried März, Rudolf A. de Boer

**Affiliations:** 1 Department of Cardiology, University Medical Center Groningen, University of Groningen, 9700 RB Groningen, The Netherlands; 2 Department of Cardiology, Thorax Center, Erasmus University Medical Center, P.O. Box 2040, 3000 CA Rotterdam, The Netherlands; 3 Mannheim Medical Faculty, Medical Clinic V (Nephrology, Hypertensiology, Endocrinology, Diabetology, Rheumatology), University of Heidelberg, 68167 Mannheim, Germany; 4 SYNLAB MVZ Humangenetik Mannheim, 68163 Mannheim, Germany; 5 Department of Experimental Hematology, University Medical Center Groningen, University of Groningen, 9700 RB Groningen, The Netherlands; 6 Synlab Academy, SYNLAB Holding Deutschland GmbH, 68159 Mannheim, Germany

**Keywords:** biomarker, galectin-3, blood group, von Willebrand factor, prognosis

## Abstract

Previous studies have reported an association between ABO type blood group and cardiovascular (CV) events and outcomes. The precise mechanisms underpinning this striking observation remain unknown, although differences in von Willebrand factor (VWF) plasma levels have been proposed as an explanation. Recently, galectin-3 was identified as an endogenous ligand of VWF and red blood cells (RBCs) and, therefore, we aimed to explore the role of galectin-3 in different blood groups. Two in vitro assays were used to assess the binding capacity of galectin-3 to RBCs and VWF in different blood groups. Additionally, plasma levels of galectin-3 were measured in different blood groups in the Ludwigshafen Risk and Cardiovascular Health (LURIC) study (2571 patients hospitalized for coronary angiography) and validated in a community-based cohort of the Prevention of Renal and Vascular End-stage Disease (PREVEND) study (3552 participants). To determine the prognostic value of galectin-3 in different blood groups, logistic regression and cox regression models were used with all-cause mortality as the primary outcome. First, we demonstrated that galectin-3 has a higher binding capacity for RBCs and VWF in non-O blood groups, compared to blood group O. Additionally, LURIC patients with non-O blood groups had substantially lower plasma levels of galectin-3 (15.0, 14.9, and 14.0 μg/L in blood groups A, B, and AB, respectively, compared to 17.1 μg/L in blood group O, *p* < 0.0001). Finally, the independent prognostic value of galectin-3 for all-cause mortality showed a non-significant trend towards higher mortality in non-O blood groups. Although plasma galectin-3 levels are lower in non-O blood groups, the prognostic value of galectin-3 is also present in subjects with a non-O blood group. We conclude that physical interaction between galectin-3 and blood group epitopes may modulate galectin-3, which may affect its performance as a biomarker and its biological activity.

## 1. Introduction

Cardiovascular (CV) diseases account for 32% of global deaths, and the prognosis of patients with CV disease, particularly in patients with heart failure, remains poor; it is, therefore, important to further investigate disease characteristics and identify risk factors that can serve as therapeutic targets [1,2]. Besides the classical risk factors of heart failure such as hypertension, smoking, dyslipidaemia, obesity, and diabetes mellitus, a sedentary habit, excessive alcohol intake, influenza, certain microbes, cardiotoxic drugs, chest radiation, and coronary artery disease also have to be considered [3,4]. However, the residual risk remains high, and we do not fully understand all factors contributing to CV disease development.

In the past years, the ABO blood group has been identified as a novel and intriguing risk factor for CV disease. Multiple studies have shown an association between non-O blood groups and the risk of different thromboembolic events [5], coronary heart disease [6], the size of a myocardial infarction after an acute coronary syndrome [7], increased mortality in patients with ischemic heart disease [8], and venous thrombosis [9]. The exact mechanisms behind these associations remain unclear to date, but as a possible common mechanism, variable levels and activity of the von Willebrand Factor (VWF) have been proposed. VWF is widely acknowledged as a key determinant in CV homeostasis and has been linked to thrombosis and CV events [10,11]. VWF was also found to be a binding partner of galectin-3 [12].

Galectin-3 is a carbohydrate-binding protein and has been shown to be involved in inflammation, cancer, and CV disease [13,14,15,16,17]. It was shown that galectin-3 is able to modulate VWF-mediated thrombus formation via a direct (physical) interaction with VWF [12]. A possible link between galectin-3 and blood group has been described previously—a genome-wide association study showed that the ABO gene locus was strongly associated with plasma galectin-3 levels [18]. This ABO locus appears to be a very pleiotropic locus that associates with several CV traits [19]. Building upon those findings, we hypothesized that ABO, galectin-3, and VWF would interact and, specifically, that the described associations between galectin-3 and CV outcome [20] can, at least partially, be explained by an interaction with the ABO blood group and VWF levels.

## 2. Results

### 2.1. Study Population

The baseline characteristics of patients in the LURIC study are presented in Table 1. The mean age (SD) was 63 (10) years, and the majority of the population was male (68%). Out of the population, 946 (37%) of the patients had blood group O, 1219 (47%) blood group A, 276 (11%) blood group B, and 130 (5%) blood group AB. Additionally, 495 (19%) of the patients were smokers, and a medical history of hypertension and coronary artery disease were very common (73% and 77%, respectively). To validate our findings, we studied the relationship between galectin-3 and the blood group in a community-based cohort, for which we used the PREVEND study. Participants of the PREVEND cohort were younger (mean age 50 ± 12), and sex was equally distributed (51% male versus 49% female). 1557 (44%) had blood group O, 1606 (45%) blood group A, 271 (8%) blood group B, and 118 (3%) blood group AB. Smoking was common (46%), but hypertension was less abundant compared to the LURIC cohort (30%), as expected (Supplemental Table S1).

### 2.2. Galectin-3 Plasma Levels Stratified by Blood Group

**Figure 1:**
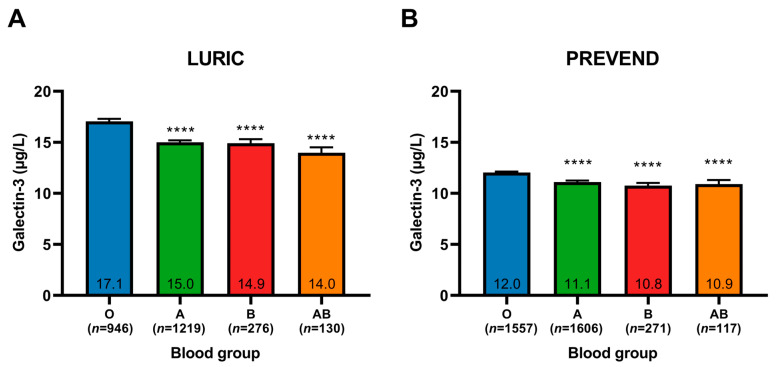
Plasma galectin-3 levels in (**A**) LURIC and (**B**) PREVEND participants, stratified by ABO blood group. Data is presented as mean ± SEM. **** *p* < 0.0001 compared to blood group O (Dunn’s multiple comparisons test).

The LURIC cohort was stratified by blood group. Plasma levels of galectin-3 were significantly higher in blood group O compared to other blood groups (*p* < 0.0001 for all groups versus blood group O) (Table 1, Figure 1A). Furthermore, VWF levels were significantly lower in blood group O compared to other blood groups (Table 1). In the PREVEND cohort, galectin-3 levels were also significantly different among blood groups and showed the highest values in blood group O compared to other blood groups (Figure 1B, Supplemental Table S1). Moreover, subjects with homozygous blood groups showed a trend towards lower plasma levels of galectin-3 compared to subjects with heterozygous blood groups (Supplemental Figure S1).

### 2.3. Binding of Galectin-3 and Red Blood Cells

**Figure 2:**
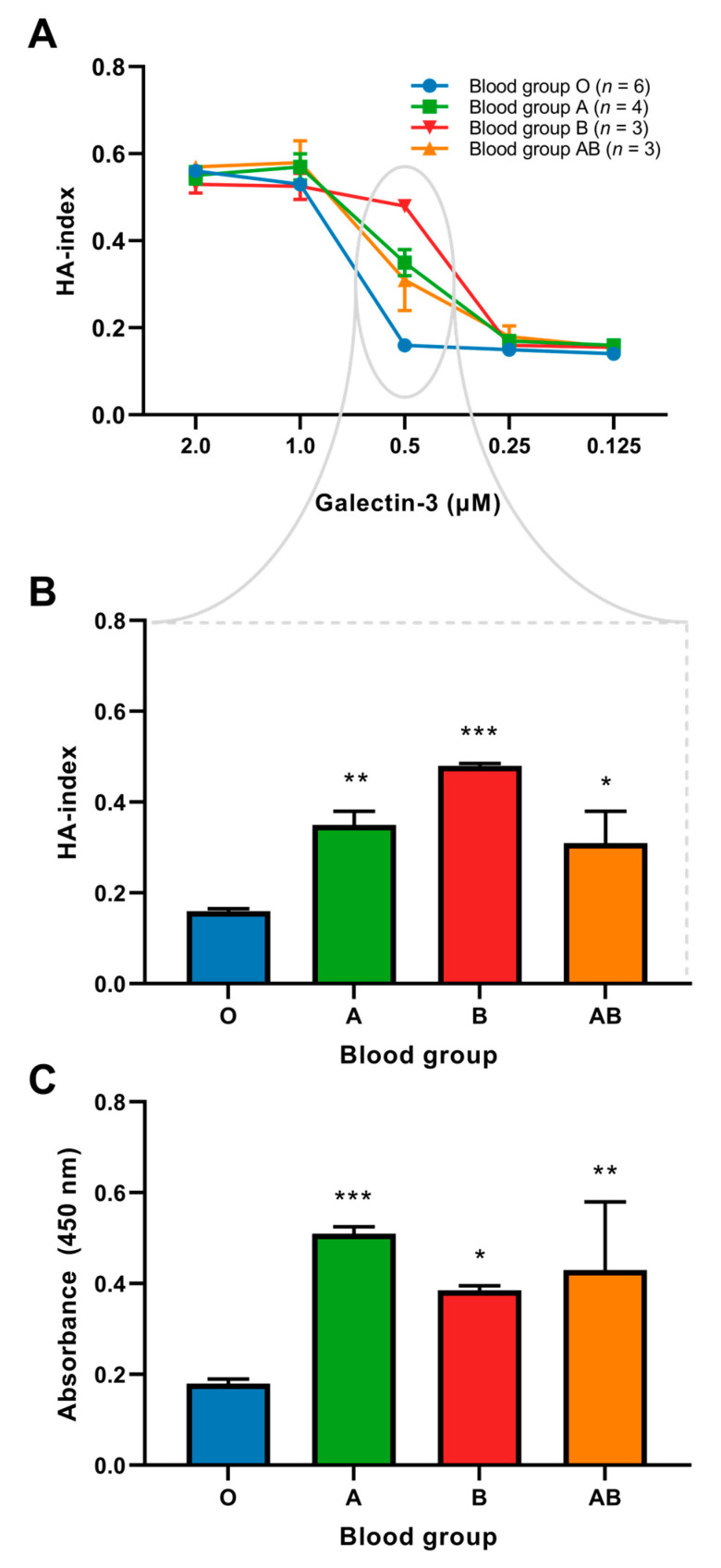
(**A**) Galectin-3-mediated hemagglutination of blood from different blood groups. (**B**) Hemagglutination induced by 0.5 µM galectin-3. (**C**) Galectin-3 binding to VWF in different blood groups, as assessed by measuring absorbance at 450 nm. * *p* < 0.05 compared to blood group O; ** *p* < 0.01 compared to blood group O; *** *p* < 0.001 compared to blood group O. Abbreviations: HA-index, hemagglutination-index.

Galectin-3 is known to mediate the hemagglutination of red blood cells (RBCs). To further characterize a potential interaction between galectin-3, VWF, and blood group, two different in vitro assays were performed. The first assay was a hemagglutination assay, to examine the interaction between galectin-3 and the blood group, as displayed in Supplemental Figure S2. With this assay we showed that the binding of galectin-3 with RBCs was significantly different between blood groups, with RBCs from blood group O binding less galectin-3 compared to all other blood groups (Figure 2A,B).

### 2.4. Interaction between VWF and Galectin-3

Since galectin-3 has been presented as a partner for VWF, we assessed the binding of VWF with galectin-3 in different blood groups using a VWF-galectin-3 binding assay. Blood plasma with similar levels of VWF (as determined with ELISA) was equalized to similar concentrations with 0.9% NaCl and incubated in a plate coated with galectin-3. Using VWF antibodies, the galectin-3-VWF binding was detected. This assay showed that the binding for galectin-3 to VWF was stronger in all non-O blood groups compared to blood group O (Figure 2C).

### 2.5. Prognostic Value of Galectin-3

**Figure 3:**
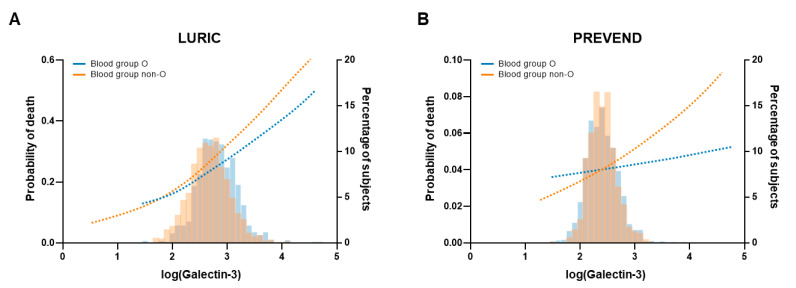
Graphical illustration of the probability of death by log-galectin-3 levels, in subjects with blood group O (blue) and blood group non-O (orange), depicted on left *y*-axis. Data are presented as adjusted splines of predicted Odds Ratio. The histograms represent the percentage of subjects with that specific log-galectin-3 level in subjects with blood group O (blue) and blood group non-O (orange), depicted on right *y*-axis. (**A**) Probability of death in LURIC cohort. Odds ratio 1.77 [1.07–2.93] and 2.35 [1.64–3.36] in blood group O and blood group non-O, *p* = 0.026 and *p* < 0.0001, respectively. (**B**) Probability of death in PREVEND cohort. Odds ratio 0.94 [0.42–2.10] and 1.48 [0.77–2.87] in blood group O and blood group non-O, *p* = 0.887 and *p* = 0.241, respectively. A similar increase in galectin-3 has more prognostic value in subjects with non-O blood group compared to subjects with blood group O. Data are adjusted for age, sex, eGFR, smoking, systolic blood pressure, BMI, LDL-cholesterol, diabetes mellitus, lipid lowering therapy, triglycerides, and CRP.

We studied the prognostic value of galectin-3 in different blood groups in the LURIC study cohort. During a median follow-up time of 9.8 [8.6–10.4] years, 758 deaths (29%) were observed. Using Cox regression analyses, galectin-3 remained a significant predictor for all-cause mortality, even after multivariate adjustment (HR 1.89 [1.28–2.79] and HR 2.19 [1.67–2.86] in blood group O and blood group non-O, respectively) (Table 2). The HR is higher in non-O blood groups, although galectin-3 plasma levels were lower in these patients (Figure 3A).

We also assessed the prognostic value of galectin-3 among different blood groups in the general population after adjustment for the same variables. In the PREVEND study, the median follow-up time was 12.6 [12.3–12.9] years, and 353 subjects (10%) died during this period. The same trend was observed compared to the LURIC study: galectin-3 appeared to have a higher prognostic value regarding all-cause mortality in non-O blood groups (Figure 3B), although the *p* for interaction was non-significant. The blood group itself was not an independent predictor for outcome in both the LURIC and PREVEND study cohorts (Supplemental Table S2).

## 3. Discussion

We demonstrate that circulating galectin-3 levels in subjects with non-O blood groups are significantly lower compared to levels in subjects with blood group O. However, the prognostic value of galectin-3 is stronger in subjects with non-O blood groups. As a potential mechanism, we propose that VWF may mediate this, as circulating VWF and galectin-3 were inversely related. We demonstrate that galectin-3 binds stronger to RBCs and VWF of subjects with non-O blood groups compared to subjects with blood group O.

Accumulating evidence suggests that the ABO blood group is involved in the pathogenesis of CV disease and that non-O blood groups had the highest risk of CV disease [19,21]. Previous studies have shown that the presence of non-O blood groups is associated with worse outcomes compared to blood group O [21,22,23,24]. In a recent case-control study consisting of 165 centenarians and 5063 blood donors from the same geographical region, it was observed that among centenarians the prevalence of blood group O was higher (56.4% vs. 43.5%; *p* = 0.001) [25]. Besides studies that demonstrate a higher CV risk for non-O blood groups, there are a few studies that specifically found the highest risk, for blood group A and blood group AB [6,21,26,27,28,29]. For example, one recent Finnish study found the highest risk of ischaemic heart disease in a patient with blood group A with T1DM and microalbuminuria [27]. A Canadian study (*n* = 64,686) demonstrated that blood group AB is associated with an increased risk of thrombotic events in participants from Quebec [28].

The ABO(H) blood group is the most important blood group system and is determined by complex carbohydrate moieties at the extracellular surface of the RBC membrane [30]. The A and B alleles encode for either A- or B-glycosyltransferases that add *N*-acetylgalactosamine or D-galactose to the common H-glycan precursor backbone, respectively. In subjects with blood group O, no A- or B-transferase activity is present, resulting in the expression of the H-glycan backbone without an additional group [31]. Next to the expression of RBCs, these blood group epitopes and different antigens are also expressed on other cells, such as the vascular endothelium, epithelial cells, T-cells, B-cells, and platelets, and present on molecules such as VWF [32,33].

Several studies described the major effects of the ABO blood group on plasma levels of VWF: plasma VWF levels appear to be 25% lower in the O blood group compared to non-O blood groups [6]. This implies that subjects with blood group O may experience a higher incidence of bleeding events, while subjects with non-O blood groups experience a higher incidence of thrombotic events [34,35]. The exact mechanisms underpinning these observations remain unclear, but this effect may be mediated by VWF. The effect of the ABO blood group on plasma levels of VWF seems to be the result of a direct effect of the ABO blood group [36]. The conversion of the blood group O determinant into other antigens of the ABO blood group was correlated with an increased capacity to modify the N-linked glycosylation of VWF [37]. Therefore, changes in VWF glycan composition also affect the biological activity of VWF and are not restricted to its plasma levels [38]. Carbohydrate structures on the surface of VWF play an important role in the life cycle of VWF. Galectin-3 is a carbohydrate-binding protein and has recently been identified as a new partner of VWF [12]. Furthermore, the affinity of transmembrane glycoproteins to the galectin-3 molecule is proportional to the number and branching of their N-glycans [39]. Therefore, we hypothesize that the biological activity of galectin-3 might also be directly regulated by the glycosylation of the molecule by the ABO blood group.

In agreement with previous studies, we confirmed that plasma VWF levels are ~25% higher in non-O blood groups. Additionally, we now show in two independent cohorts with different populations, that galectin-3 levels are significantly lower in non-O blood groups. Furthermore, we show that galectin-3 levels are lower in patients who had a heterozygous blood group. This inverse relationship between galectin-3 and VWF levels in different blood groups is an interesting phenomenon, potentially explained by the fact they are ligands of each other.

Numerous studies have assessed the prognostic value of galectin-3 in various cohorts [40,41,42]. We again corroborated these findings in the current study and herein confirm that galectin-3 is an independent predictor for all-cause mortality, particularly in subjects with non-O blood groups. The striking observation that galectin-3 has a strong prognostic value in non-O blood groups, although the group has lower galectin-3 values, should be explored in further detail. We speculate that the observed lower galectin-3 plasma values in the non-O blood group participants are caused by galectin-3 binding with blood group epitopes and that glycosylation might play a role in this.

In two different in vitro assays, we show a higher binding capacity of galectin-3 with RBCs and VWF in subjects with non-O blood groups, compared to blood group O. Binding preference of galectin-3 is most likely related to the extensive glycosylation of VWF, generating a clustered glycan surface, resembling the cell membrane [12]. These protein-glycan interactions between VWF and galectin-3 mainly consist of binding patterns with *N*-linked glycans rather than *O*-linked glycans, as has been shown previously [43]. Galectins regularly show a high affinity for glycans with longer poly-*N*-acetyllactosamine (poly-LacNAc) chains, given their higher binding capacity for *N*-linked glycans.

The higher hemagglutination activity in subjects with non-O blood groups is consistent with previous findings from erythrocyte binding and glycan microarray studies, suggesting that galectin-3 exhibits higher binding towards blood group A and B antigens compared to those bearing the H antigen [43,44,45,46]. While all galectins show a high affinity for β-galactosides, their recognition following terminal glycan modifications varies. The enhanced recognition of galectin-3 towards A and B blood group substitutions is potentially caused by unique subsides within the carbohydrate recognition domain (CRD) [43] and might play an evolutionary role. In fact, it enables the targeting of microbes that utilize blood group molecular mimicry [47]. Additionally, we hypothesize that stronger binding of galectin-3 with RBCs and VWF in non-O blood groups could explain lower levels of circulating galectin-3.

The prognostic value and absolute levels of biomarkers may differ between different subgroups in a study cohort, as previously observed for other biomarkers [48]. For instance, plasma levels differ between sexes, and also age, renal function, and the presence of diabetes are important determinants of hemoglobin level [49,50]. Even for the established cardiac marker NT-proBNP, important determinants exist leading to differences in circulating levels; renal failure tends to increase natriuretic peptide levels, whereas patients with obesity show lower levels of NT-proBNP [51,52]. Using a combination of biomarkers might improve risk prediction of clinical outcomes and, therefore, healthcare-related costs.

In conclusion, we postulate that the binding of galectin-3 to the A-, B-, and AB- blood group epitopes affects the circulating plasma levels and its biological activity, and thereby also its prognostic power for a given concentration. Future studies should provide more detailed data on this interaction and practical information on how to deal with this potential confounder.

## 4. Materials and Methods

### 4.1. Study Population

#### 4.1.1. LURIC

The Ludwigshafen Risk and Cardiovascular Health (LURIC) study consists of 3316 patients who were hospitalized for coronary angiography between 1997 and 2000. Indications for coronary angiography were chest pain or a positive non-invasive stress test suggestive of myocardial ischemia. Further methods and results have been described previously [53]. In total, galectin-3 values and blood group information were available for 2571 patients.

#### 4.1.2. PREVEND

The Prevention of Renal and Vascular End-stage Disease (PREVEND) study is a prospective, observational, community-based study and was used to validate our findings [18,54]. The PREVEND study enrolled community-dwelling subjects during 1997–1998, and the study was designed to track the long-term development of cardiac, renal, and peripheral vascular disease. More details of the design of the study have been described previously [55,56]. Galectin-3 and blood group data were available in 3552 subjects.

In both studies, all participants provided informed consent, and the study procedures were conducted in accordance with the 1975 Declaration of Helsinki. The LURIC study was approved by the ethical committee of the Ärztekammer Rheinland-Pfalz, and the PREVEND study was approved by the ethical committee of the University Medical Center Groningen (UMCG).

### 4.2. Galectin-3 Measurements

In the LURIC study, galectin-3 levels were measured in plasma samples from the baseline. These samples were stored at −80 °C and were analysed using the ARCHITECT analyser (Abbott Diagnostics, Abbott Park, IL, USA). This automated assay uses the same antibodies and conjugates as in the manual assay and has a lower limit of detection of 1.01 ng/mL. Intra- and inter-assay variability are 3.2% and 0.8%, respectively [57]. In the PREVEND study, blood was drawn at the baseline and anticoagulated with EDTA. Samples were stored at −80 °C until the time of analysis. Galectin-3 concentration was measured in plasma samples from the baseline using the BGM galectin-3 ELISA kit (BG Medicine Inc., Waltham, MA, USA). Intra- and inter-assay coefficients of this assay are 3.2% and 5.6%, respectively. The assay has a lower limit of detection of 1.13 ng/mL and did not show cross-reactivity with collagens or other members of the galectin family [58].

### 4.3. Blood Group Determination

Blood group in LURIC was determined in the Haemostaseology Laboratory of the Ludwigshafen Cardiac Centre using a blood group antisera macroscopic agglutination assay (ABO- and Rh-blood group sera, Loxo GmbH, Dossenheim, Germany). In the PREVEND cohort, the ABO blood group was inferred from genotyping three single nucleotide polymorphisms (SNPs) on the ABO gene, namely rs8176719, rs8176746, and rs8176747. Using a combination of these SNPs, a blood group could be determined, as described previously [59].

### 4.4. Clinical Endpoints

In LURIC, mortality data were collected from local registries. Two independent and experienced clinicians, who were blinded for patient characteristics, reviewed information from death certificates, medical records from hospitals, and data from autopsies [20,60]. In PREVEND, mortality data were collected using the municipal register, and cause of death was obtained using the Prismant health care data system or Dutch Central Bureau of Statistics. Follow-up times ranged from the last follow-up or were censored on the date of the event or last contact, whatever occurred first.

### 4.5. In Vitro Studies

#### 4.5.1. Isolation of Red Blood Cells

Neonatal cord blood was obtained from healthy full-term pregnancies from donors from the obstetrics departments of the Martini Hospital Groningen and UMCG after informed consent was given. All donors were informed about the studies that were performed, as approved by the local Medical Ethical Committee of the UMCG. Furthermore, healthy volunteers from the research lab also provided blood specimens. Blood was collected in 10 mL EDTA tubes and 20 µL of blood was used to determine the ABO blood group using a Serafol ABO bedside test (Bio-Rad Laboratories BV, Veenendaal, the Netherlands). The remaining blood was centrifuged at 3500 rpm for 5 min. The buffy coat appeared as a dense white layer in the middle between the RBCs and plasma. Plasma and the buffy coat were removed from the tube. RBCs remained in the tube and were resuspended in PBS and again centrifuged at 2000 rpm for 5 min at 4 °C. This washing step was repeated 3 times. Subsequently, the remaining RBCs were diluted 12.5× in PBS-3% glutaraldehyde in a tube, and this was put on a rotating wheel for 1 h at room temperature. Afterwards, the cells were washed 5 times with PBS (0.0025% NaN_3_) and centrifuged at 2000 rpm for 2 min at 4 °C, and in the last step, cells were resuspended at 3–4% in PBS (0.0025% NaN_3_). Cells were stored at 4 °C for several days.

#### 4.5.2. Hemagglutination Assay

RBCs were counted using a Fuchs-Rosenthal counting chamber. All cells were diluted to the lowest concentration of RBCs. We first calibrated our hemagglutination assay to determine the number of RBCs that were needed to show hemagglutination and to clearly distinguish between agglutinated and non-agglutinated cells. We tested 3 different concentrations of RBCs (5 µL/10 µL/15 µL of 2000 cells/µL) and 2 concentrations of galectin-3 (1 µM/2 µM). Following calibration, we used 15 µL RBCs/2 µM galectin-3 in the first well of a round-bottom, 96-well plate (Costar #3799, Corning Inc., Kennebunk, ME, USA). Next, 2 µM galectin-3 was serially diluted 1:1 into the next wells and 87,5 µL PBS was added to a total volume of 185 µL. Finally, 15 µL (2000 cells/µL) of RBCs were added to each well. The plate was incubated for 30 min at 4 °C and pictures were made using the ImageQuant LAS 4000 (GE Healthcare, Europe GmbH, Diegem, Belgium). Hemagglutination was assessed using ImageJ software (Version 1.50, National Institutes of Health, Bethesda, MD, USA), and the hemagglutination-index ((surface area of RBCs after incubation/surface area of the total well) × 100) (HA-index) was calculated.

#### 4.5.3. Von Willebrand Factor ELISA

VWF was measured in human plasma using the VWF ELISA kit (Abcam, Cambridge, UK). This kit was designed for the quantitative measurement of human VWF in plasma, serum, and cell culture supernatants. Intra- and inter-assay coefficients of variation of this assay are 5% and 7.1%, respectively. The lower level of detection is 2.5 mU/mL.

In LURIC, VWF was measured using the STA Liatest^®^VWF assay (Stago Diagnostica/Roche, Mannheim, Germany).

#### 4.5.4. Galectin-3—von Willebrand Factor Binding Study

As previously described [12], an immunosorbent assay was performed in which a microtiter 96-well plate was coated with galectin-3 (5 µg/well) overnight at 4 °C. After washing 3 times with PBS (0.1% Tween-20) the plate was blocked for 2 h with PBS (0.1% Tween-20/3% BSA) at 37 °C. After washing 2 times with PBS (0.1% Tween-20), plasma of different blood groups was incubated in the wells for 1 h at 37 °C. After discarding the plasma, the plate was washed 2 times with PBS (0.1% Tween-20). Bound VWF was detected by adding 50 µL HRP-labelled polyclonal VWF antibody (1:1000; P0226, DAKO, Glostrup, Denmark). 50 µL 3,3′,5,5′-tetramethylbenzidine (TMB) was added to detect HRP activity, and after 10 min 50 µL of stop solution (H_2_SO_4_) was added to stop the reaction. The absorbance was measured using a microplate reader at a wavelength of 450 nm (BioTek Synergy H1, Winooski, VT, USA).

### 4.6. Statistical Analysis

Normally distributed variables are presented as means ± standard deviation (SD) or standard error of the mean (SEM). Non-normally distributed variables are expressed as medians [interquartile range (IQR)]. To compare normally distributed values across two groups, a two-sample *t*-test was performed, and to compare non-normally distributed values, we used the Wilcoxon rank-sum test. The comparison of categorical values was done using Pearson’s Chi-square test. Characteristics across four groups were compared using the ANOVA for continuous and normally distributed values and the Kruskal-Wallis test for continuous, non-normally distributed values. In a comparison of >1 group with a control group, we used the Kruskal-Wallis with a post hoc Dunn’s multiple comparisons tests.

Prior to analysis, galectin-3 was transformed logarithmically to obtain approximately normal distributions because of a skewed distribution as assessed by the Shapiro-Wilk test. To study the association of galectin-3 with all-cause mortality, Cox regression analysis and logistic regression analysis were performed with log-transformed galectin-3 as a continuous variable. The model was adjusted for age and sex and a multivariable model consisting of eGFR, smoking, systolic blood pressure, BMI, LDL-cholesterol, diabetes mellitus, lipid-lowering therapy, triglycerides, and CRP. This model is an established risk model for all-cause mortality in the LURIC study and has been used previously in other studies [20]. Results are stratified to blood group and summarized as hazard ratios, with 95% confidence intervals (CI). For the interaction term, a *p*-value of <0.10 was considered to indicate statistical significance. For all other analyses, *p*-values <0.05 were considered to be statistically significant. Analyses were performed using STATA software version 14.2 and GraphPad Prism version 9.3.1 (GraphPad Software Inc., La Jolla, CA, USA).

## Figures and Tables

**Table 1:** Baseline characteristics of the LURIC study participants, stratified by ABO blood group.

Clinical Characteristics	Total	Blood Group O	Blood Group A	Blood Group B	Blood Group AB	*p*-Value
	(*n* = 2571)	(*n* = 946)	(*n* = 1219)	(*n* = 276)	(*n* = 130)	
Age (y), mean (SD)	63 (10)	63 (10)	63 (11)	63 (11)	62 (11)	0.68
Male sex, *n* (%)	1756 (68)	642 (68)	842 (69)	187 (68)	85 (65)	0.81
Current smoker, *n* (%)	495 (19)	201 (21)	211 (17)	57 (21)	26 (20)	0.12
BMI (kg/m^2^), mean (SD)	278 (4)	28 (4)	27 (4)	27 (4)	27 (3)	0.08
Heart rate (bpm), median [IQR]	68 [61–75]	68 [61–75]	68 [61–76]	66 [60–74]	66 [60–76]	0.40
Systolic blood pressure, median [IQR]	140 [123–157]	142 [123–158]	140 [124–156]	140 [123–158]	136 [121–152]	0.30
Diastolic blood pressure, median [IQR]	81 [73–88]	81 [73–89]	80 [73–88]	81 [73–90]	80 [72–89]	0.89
**Medical history, *n* (%)**						
Type 2 diabetes mellitus	1032 (40)	372 (39)	507 (42)	104 (38)	49 (38)	0.50
(History of) hypertension	1865 (73)	691 (73)	880 (72)	200 (72)	91 (70)	0.90
(History of) coronary artery disease	1986 (77)	720 (76)	949 (78)	216 (78)	96 (74)	0.59
**Laboratory measurements**						
eGFR (MDRD), median [IQR]	81 [69–92]	80 [68–91]	81 [70–93]	80 [70–92]	81 [70–92]	0.11
Glucose (mmol/L), median [IQR]	5.7 [5.2–6.6]	5.7 [5.2–6.6]	5.7 [5.2–6.6]	5.7 [5.2–6.6]	5.6 [5.2–6.3]	0.62
Cholesterol (mmol/L), median [IQR]	4.9 [4.3–5.6]	4.9 [4.2–5.5]	4.9 [4.3–5.7]	4.9 [4.4–5.6]	4.8 [4.2–5.4]	0.25
LDL (mmol/L), median [IQR]	2.9 [2.4–3.6]	2.9 [2.4–3.5]	3.0 [2.4–3.6]	3.0 [2.5–3.6]	2.9 [2.3–3.5]	0.16
HDL (mmol/L), median [IQR]	1.0 [0.8–1.2]	1.0 [0.8–1.2]	1.0 [0.8–1.2]	1.0 [0.8–1.2]	1.0 [0.8–1.2]	0.39
Triglycerides (mmol/L), median [IQR]	3.8 [2.8–5.2]	3.9 [2.8–5.3]	3.7 [2.7–5.1]	3.7 [2.8–5.2]	3.6 [2.6–5.0]	0.28
NT-proBNP (ng/L), median [IQR]	296 [108–884]	286 [104–895]	299 [109–907]	345 [112–873]	292 [104–733]	0.70
Galectin-3 (µg/L), mean (SD)	15.7 (7.0)	17.1 (7.4)	15.0 (6.7)	14.9 (6.6)	14.0 (6.1)	<0.001
VWF (U/dL), median [IQR]	156 [120–198]	132 [100–176]	165 [130–206]	176 [136–214]	189 [145–228]	<0.001

Abbreviations: BMI, body mass index; bpm, beats per minute; eGFR, estimated glomerular filtration rate; IQR, interquartile range; HDL, high-density lipoprotein; LDL, low-density lipoprotein; MDRD, Modification of Diet in Renal Disease; NT-proBNP, N-terminal pro-B-type natriuretic peptide; SD, standard deviation; VWF, von Willebrand Factor.

**Table 2:** Cox proportional hazard analyses of log-transformed galectin-3 for the risk on all-cause mortality, divided by blood group.

LURIC Study		Blood Group O	*p*-Value	Blood Group Non-O	*p*-Value	*p*-Value for Interaction
All-cause mortality		HR [95% CI]		HR [95% CI]		
Galectin-3		2.73 [2.03–3.67]	<0.001	3.06 [2.45–3.83]	<0.001	0.53
Galectin-3		2.31 [1.65–3.24]	<0.001	2.20 [1.73–2.80]	<0.001	0.78
+sex & age						
Galectin-3		1.89 [1.28–2.79]	0.001	2.19 [1.67–2.86]	<0.001	0.89
+fully adjusted *						
**PREVEND Study**		**Blood Group O**	***p*-Value**	**Blood Group Non-O**	***p*-Value**	***p*-Value for Interaction**
All-cause mortality		HR [95% CI]		HR [95% CI]		
Galectin-3		2.48 [1.70–3.63]	<0.0001	3.44 [2.45–4.84]	<0.0001	0.20
Galectin-3		1.31 [0.74–2.32]	0.35	1.87 [1.16–3.03]	0.010	0.28
+sex & age						
Galectin-3		1.05 [0.53–2.09]	0.87	1.44 [0.83–2.48]	0.20	0.82
+fully adjusted *						

* Adjusted for age, sex, eGFR, smoking, systolic blood pressure, BMI, LDL-cholesterol, diabetes mellitus, lipid lowering therapy, triglycerides, and CRP. Abbreviations: CI, confidence interval; HR, hazard ratio.

## Data Availability

Data will be made available upon request.

## Supplementary Materials

The following supporting information can be downloaded at: https://www.mdpi.com/article/10.3390/ijms24054415/s1.
