# Supplementary material for: Galectin-3 and Blood Group: Binding Properties, Effects on Plasma Levels, and Consequences for Prognostic Performance
Source: Int J Mol Sci. 2023 Feb 23;24(5):4415. doi: 10.3390/ijms24054415 (PMC10002292; doi:10.3390/ijms24054415)

Supplemental Table S1. Baseline characteristics of the PREVEND study participants, stratified by ABO blood group.

**Clinical characteristics**	**Total** (*n*=3552)	**Blood group O** (*n*=1557)	**Blood group A** (*n*=1,606)	**Blood group B** (*n*=271)	**Blood group AB** (*n*=118)	***p*-value**
Age (y), mean (SD)	50 (12)	49 (12)	50 (13)	51 (12)	49 (12)	0.28
Male sex, *n* (%)	1,812 (51)	794 (51)	830 (52)	125 (46)	63 (53)	0.35
Smoked last 5 years, *n* (%)	1,610 (45)	720 (46)	730 (45)	115 (42)	45 (38)	0.33
BMI (kg/m^2), mean (SD)	26 (4)	26 (4)	26 (4)	26 (4)	26 (4)	0.28
Heart rate (bpm), median [IQR]	68 [62-75]	68 [62-75]	68 [62-75]	70 [64-77]	70 [62-78]	0.051
Systolic blood pressure, median [IQR]	126 [114-141]	125 [113-141]	126 [114-141]	126 [113-145]	124 [116-140]	0.85
Diastolic blood pressure, median [IQR]	73 [67-80]	73 [67-81]	73 [67-80]	75 [67-81]	75 [69-80]	0.53
**Medical history, *n* (%)**						
Type 2 diabetes mellitus, *n* (%)	53 (1)	25 (2)	21 (2)	4 (1)	3 (3)	0.71
(History of) hypertension, *n* (%)	1,057 (30)	460 (30)	488 (30)	72 (27)	37 (31)	0.28
**Laboratory measurements**						
eGFR, median [IQR]	97 [85-107]	96 [84-107]	97 [85-107]	97 [81-107]	95 [88-109]	0.86
Glucose (mmol/L), median [IQR]	4.7 [4.3-5.1]	4.7 [4.3-5.1]	4.7 [4.4-5.2]	4.7 [4.4-5.0]	4.7 [4.3-5.1]	0.52
Cholesterol (mmol/L), median [IQR]	5.6 [4.9-6.4]	5.5 [4.9-6.3]	5.6 [4.9-6.4]	5.6 [4.9-6.3]	5.6 [4.9-6.5]	0.29
LDL (mmol/L), median [IQR]	3.6 [3.0-4.3]	3.3 [2.7-4.2]	3.5 [2.8-4.2]	3.4 [2.6-4.0]	3.8 [2.8-4.1]	0.52
HDL (mmol/L), median [IQR]	1.3 [1.0-1.5]	1.3 [1.0-1.5]	1.3 [1.0-1.5]	1.3 [1.1-1.6]	1.3 [1.0-1.5]	0.58
Triglycerides (mmol/L), median [IQR]	1.2 [0.8-1.7]	1.2 [0.8-1.7]	1.2 [0.9-1.7]	1.1 [0.8-1.7]	1.4 [0.9-1.9]	0.14
NT-proBNP (ng/L), median [IQR]	37.2 [16.5-73.7]	36.5 [16.2-74.8]	36.9 [16.1-72.4]	41.8 [20.0-78.7]	33.8 [15.0-62.4]	0.27
Galectin-3 (µg/L), mean (SD)	11.5 (4.0)	12.0 (3.9)	11.1 (3.7)	10.8 (4.4)	10.9 (4.4)	<0.001

Abbreviations: BMI, body mass index; bpm, beats per minute; eGFR, estimated glomerular filtration rate; HDL, high-density lipoprotein; LDL, low-density lipoprotein; NT-proBNP, N-terminal pro-B-type natriuretic peptide.

Supplemental Table S2: Hazard ratio [95% CI] for all-cause mortality, divided by blood group, using blood group O as the reference group, in the LURIC and PREVEND cohort.

	**LURIC study**		**PREVEND study**	
	HR [95% CI]	*p*-value	HR [95% CI]	*p*-value
**Blood group:**				
O	1.00		1.00	
non-O	1.08 [0.93-1.26]	0.296	1.13 [0.92-1.40]	0.250
+ Adjusted for age, sex				
O	1.00		1.00	
non-O	1.06 [0.92-1.24]	0.386	1.07 [0.87-1.33]	0.510
+ Fully adjusted*				
O	1.00		1.00	
non-O	1.06 [0.91-1.23]	0.461	1.02 [0.81-1.29]	0.857

* Adjusted for age, sex, eGFR, smoking, systolic blood pressure, BMI, LDL-cholesterol, diabetes mellitus, lipid lowering therapy, triglycerides, CRP.

Supplemental Figure S1. Plasma galectin-3 levels in PREVEND participants, stratified by ABO blood group.

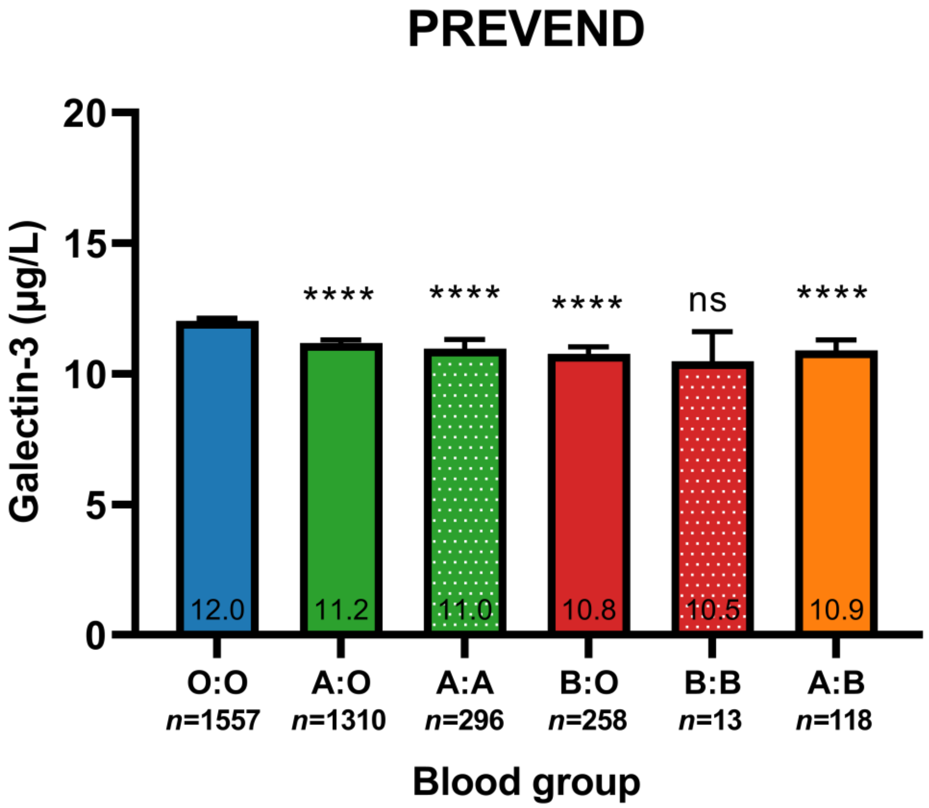

Supplemental Figure S2. Example of a hemagglutination assay.

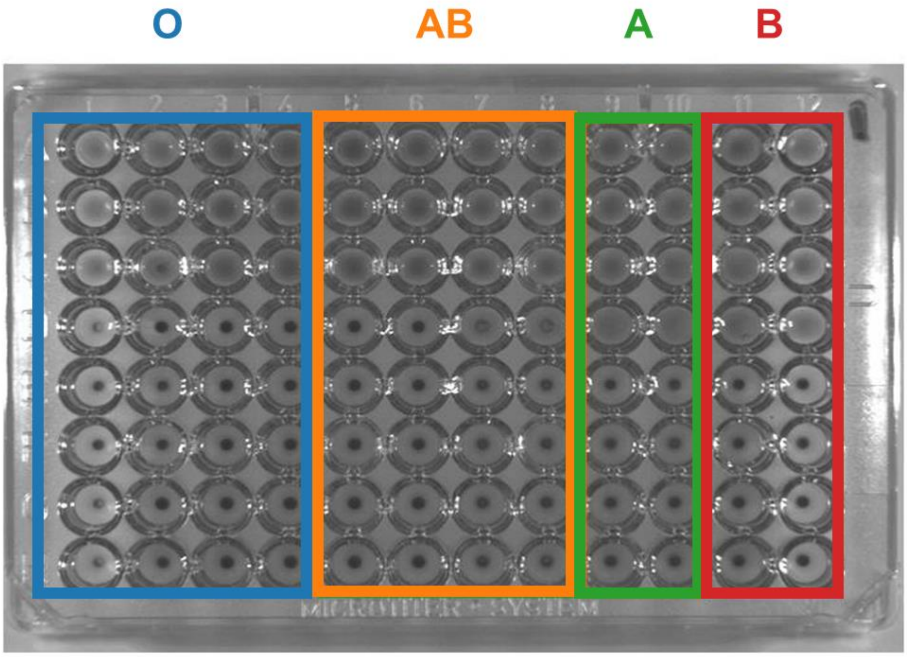

Supplement: Supplementary file 1 [file ijms-24-04415-s001.zip › ijms-2149149-supplementary.pdf]
